# Evaluation of [^89^Zr]Zr-girentuximab PET imaging of clear cell renal cell carcinoma in Chinese patients: a Phase 1 clinical study (ZIRDOSE-CP)

**DOI:** 10.1186/s13550-025-01332-5

**Published:** 2025-12-24

**Authors:** Chen Liu, Yongpeng Ji, Xiaoyan Wu, Brenda Cerqueira, David Cade, Peng Du, Zhi Yang

**Affiliations:** 1 Key Laboratory of Carcinogenesis and Translational Research (Ministry of Education), Key Laboratory of Research, Investigation and Evaluation of Radiopharmaceuticals, Department of Nuclear Medicine, NMPA Key Laboratory for Research and Evaluation of Radiopharmaceuticals (National Medical Products Administration), Department of Nuclear Medicine Peking University Cancer Hospital & Institute, Beijing, 100142 China; 2 Key Laboratory of Carcinogenesis and Translational Research (Ministry of Education), Department of Urology, Peking University Cancer Hospital & Institute, Beijing, China; 3 Grand Pharmaceutical (China), Wuhan, China; 4 Telix Pharmaceuticals, North Melbourne, Australia

**Keywords:** Renal cell carcinoma, Carbonic anhydrase IX, Positron emission tomography, Radiopharmaceuticals, [^89^Zr]Zr-girentuximab

## Abstract

**Background:**

Results from a multinational Phase 3 study demonstrated high accuracy and a favorable safety and tolerability profile of [^89^Zr]Zr-girentuximab PET for detection and characterization of clear cell renal cell carcinoma (ccRCC). However, [^89^Zr]Zr-girentuximab has not been studied in Chinese patients. In this Phase 1 study, we aimed to evaluate safety and tolerability, radiation and tumor dosimetry, and pharmacokinetics of [^89^Zr]Zr-girentuximab PET in Chinese patients with suspected ccRCC. This study was approved by the Ethics Committee of Beijing Cancer Hospital (2022YW225) and was conducted in accordance with the Declaration of Helsinki. All patients provided written informed consent. Ten Chinese patients received 37 MBq (± 10%; 10mg mass dose) of [^89^Zr]Zr-girentuximab intravenously. Whole-body PET imaging was performed in the supine position at approximately 0.5, 4, 24, 72 hours, and 7 ± 1 days after administration, and low dose CT was used for attenuation correction. Adverse events were recorded from time of [^89^Zr]Zr-girentuximab administration through final visit. Blood samples were collected from patients approximately 1 hour before, and 0.5, 1, 2, 4, 24, 72 hours, and 5 ± 1 days after [^89^Zr]Zr-girentuximab administration. Biodistribution and normal organ dosimetry were performed based on PET images to determine significant differences in tumor-absorbed dose.

**Results:**

Of 10 adverse events reported, none were serious or considered related to [^89^Zr]Zr-girentuximab. The organs receiving the highest mean (SD) normalized absorbed doses were the liver (1.51 [0.23] mGy/MBq), kidneys (1.42 [0.35] mGy/MBq), and heart wall (1.22 [0.18] mGy/MBq), with a mean whole-body normalized effective dose of 0.49 (0.08) mSv/MBq. In 7 patients with histologically confirmed ccRCC, mean (SD) tumor normalized absorbed dose was 2.87 (1.54) mGy/MBq (range: 1.17 to 5.41 mGy/MBq).

**Conclusion:**

[^89^Zr]Zr-girentuximab has a favorable safety and tolerability profile in Chinese patients, with a dosimetry profile similar to previously studied non-Chinese populations. [^89^Zr]Zr-girentuximab is a promising novel PET agent for the detection and characterization of ccRCC in Chinese patients. Trial registration: ClinicalTrials.gov Identifier NCT05861778. Registered 17 May 2023.

**Supplementary Information:**

The online version contains supplementary material available at 10.1186/s13550-025-01332-5.

## Background

In China, the age-standardized incidence rate of kidney cancer is approximately 3.3 per 100,000 people, lower than the global rate of 4.5 per 100,000 [1]. The incidence of kidney cancer has been steadily increasing in China, rising at a faster rate than the global average [1]. Clear cell renal cell carcinoma (ccRCC) is the most common type of renal cell carcinoma (RCC), accounting for up to 85% of RCCs and up to 90% of RCC deaths globally [2–7]. Among patients with small renal masses on active surveillance, the ccRCC subtype is reported to progress more rapidly compared to other RCC subtypes [2, 3, 5].

While the incidence of kidney cancer is increasing globally (3.9 per 100,000 in 1990 to 4.5 per 100,000 in 2021) [1] due to factors such as improved imaging techniques and lifestyle changes [1, 8, 9], technological advancements in early detection and treatment selection remain limited [10–19]. Moreover, the currently available diagnostic options for ccRCC have limitations. Renal mass biopsy is invasive, with approximately 8% of patients undergoing biopsy for suspected RCC exhibiting biopsy-related complications [20]. Additionally, approximately 20–30% of renal mass biopsies yield a benign diagnosis [21], and up to 15% are nondiagnostic due to the heterogeneity of tumors and insufficient tissue samples [20, 22]. Consequently, noninvasive modalities are needed to detect and elucidate tumor biology, which can help risk stratify patients and support treatment decisions while minimizing invasive and potentially unnecessary biopsy or surgery.

Computed tomography (CT) and magnetic resonance imaging (MRI) may be used to characterize renal masses [17]. However, the sensitivity for predicting ccRCC in patients with suspicious small renal masses of both CT and MRI are moderate (79.7% and 88.1%, respectively), while specificity may be as low as 44% for CT and 33% for MRI [23]. Positron emission tomography (PET) with commonly used tracers such as [^18^F]F-FDG is not a standard imaging modality in patients with ccRCC due to poor uptake, low specificity and sensitivity, and renal excretion [16, 24]. PSMA PET tracers used in prostate cancer such as [^68^Ga]Ga-PSMA and [^18^F]F-PSMA have limited use in primary ccRCC due to variable PSMA expression in ccRCC, poor tumor visualization given high physiological PSMA uptake in surrounding renal tissue and renal elimination [25–27].

Girentuximab is a monoclonal antibody (mAb) that targets carbonic anhydrase IX (CAIX), a tumor-associated antigen that is highly expressed by ccRCC [28, 29]. Previous studies have demonstrated promising results with girentuximab labelled with zirconium-89 (^89^Zr; [^89^Zr]Zr-girentuximab) [30, 31]. The recent ZIRCON Phase 3 study [32] evaluated the detection and characterization of [^89^Zr]Zr-girentuximab PET imaging for ccRCC, using histology as standard of truth. Of 284 evaluable patients with indeterminate renal masses (IRMs) ≤ 7 cm in diameter, the mean sensitivity and specificity across all 3 readers was 86% (95% CI: 82%, 90%) and 87% (95% CI: 81%, 93%), respectively. The study also demonstrated high sensitivity and specificity for detection and characterization of small renal masses ≤ 4 cm and ≤ 2 cm in diameter. The clinical decision-making impact of [^89^Zr]Zr-girentuximab PET has also been demonstrated in published patient case reports including from real-world experience, demonstrating its utility for guiding surgical planning, providing confidence in treatment plan, and avoiding unnecessary interventions [33–35].

While [^89^Zr]Zr-girentuximab has been studied extensively, existing data are primarily derived from Western and Japanese populations, with limited evidence specific to Chinese patients. Of note, the genomic profile of ccRCC may vary between Chinese patients and Western counterparts due to genetic diversity across geographic regions and differences in biodistribution [36–39]. We present results from an open-label, single-center, single-arm, prospective Phase 1 study (ZIRDOSE-CP; ClinicalTrials.gov, NCT05861778) that evaluated the safety, pharmacokinetics (PK), biodistribution, and dosimetry of [^89^Zr]Zr-girentuximab PET in Chinese patients with suspected ccRCC.

## Materials & methods

### Study design and procedure

This single-center, prospective Phase 1 study (ClinicalTrials.gov: NCT05861778) was approved by the Ethics Committee of Beijing Cancer Hospital (2022YW225) and was conducted in accordance with the Declaration of Helsinki. All patients provided written informed consent before participation in the study.

The primary objective of the study was to evaluate the safety and tolerability of [^89^Zr]Zr-girentuximab. Secondary objectives included analysis of radiation and tumor dosimetry, and PK of [^89^Zr]Zr-girentuximab.

Chinese patients ≥ 18 years of age with an expected survival of ≥ 6 months and with IRMs, suspected renal cell carcinoma, or previously diagnosed ccRCC with suspected recurrence were enrolled. Eligible patients had confirmed imaging evidence (i.e., pre-screening MRI or CT) of an IRM or suspected ccRCC recurrence within 90 days before [^89^Zr]Zr-girentuximab administration. Key exclusion criteria included an IRM known to be a metastasis of another primary tumor; other malignancies requiring treatment; chemotherapy, radiotherapy, or immunotherapy within 4 weeks before planned [^89^Zr]Zr-girentuximab administration; antineoplastic therapies planned between [^89^Zr]Zr-girentuximab administration and imaging; renal insufficiency with glomerular filtration rate ≤ 60 mL/min/1.73 m^2^ within 30 days before planned [^89^Zr]Zr-girentuximab administration; or exposure to murine or chimeric antibodies within the past 5 years.

## [^89^Zr]Zr-girentuximab administration and PET/CT imaging

Patients were intravenously administered a single dose of [^89^Zr]Zr-girentuximab (37 MBq ± 10%; 10 mg girentuximab) intravenously as a slow injection over 3–5 min. Radiolabeling was conducted at JFE Engineering, a GMP-certified Contract Manufacturing Organization. The radiolabeling process involved conjugation of girentuximab with the chelator desferrioxamine-tetrafluorophenol (DFO-TFP) to produce the intermediate ^89^Zr-DFO-girentuximab. The study dose consisted of 37 MBq of ^89^Zr conjugated to girentuximab, diluted with unconjugated girentuximab in 0.9% sodium chloride to achieve a total antibody dose of 10 mg/10 mL for intravenous administration. Radiochemical purity of [^89^Zr]Zr-girentuximab was confirmed to be ≥ 90% by both instant thin-layer chromatography and size-exclusion high-performance liquid chromatography.

Whole-body PET imaging was performed in the supine position at approximately 0.5, 4, 24, 72 h, and 7 ± 1 days after administration, and low dose CT was used for attenuation correction. All PET/CT scans were performed on a UEXPLORER (serial number F00014; United Imaging, Shanghai, China). PET data were acquired in 3D list-mode and reconstructed using an ordered-subset expectation maximization algorithm (3 iterations, 20 subsets; matrix size 192 × 192; pixel size 3.125 × 3.125 mm; slice thickness 2.886 mm, no overlap). PET acquisition duration was 10 min for 1 bed position on the total-body scanner. Low-dose CT was acquired at 120 kVp with a spiral pitch factor of 1.0125, revolution time 0.5 s, and a collimation width of 0.5 mm (total collimation 40 mm). CT images were reconstructed with a slice thickness of 3 mm (50% overlap) using a 512 × 512 matrix (pixel size 0.977 × 0.977 mm). Standard quantitative corrections for normalization, randoms, dead time, decay, attenuation, and scatter, and time-of-flight and point spread function modeling were applied to all PET/CT imaging data.

## Assessments

Adverse events (AEs) were recorded from time of [^89^Zr]Zr-girentuximab administration through final visit. AEs were coded using MedDRA 26.0. The severity of AEs was graded according to National Cancer Institute-Common Toxicity Criteria for Adverse Events version 5.0. Baseline abnormality was defined as any untoward medical condition in a patient before [^89^Zr]Zr-girentuximab administration. AEs were defined as any adverse event that newly occurred or worsened after [^89^Zr]Zr-girentuximab administration. AEs were defined as not related to [^89^Zr]Zr-girentuximab by investigators if: (a) the event is more likely to be caused by the patient’s clinical status or other treatment methods or (b) the event occurred prior to administration of [^89^Zr]Zr-girentuximab or was unlikely to be related to study participation (e.g., impaired in a collision). Blood samples were collected from all patients approximately 1 h before, and 0.5, 1, 2, 4, 24, 72 h and 5 ± 1 days after [^89^Zr]Zr-girentuximab administration. Radioactivity was quantified in all blood samples.

Software used for image evaluation and dosimetry analysis was QDOSE^®^ v2.2.4 with OLINDA/EXM v2.2 [40] and IDAC-Dose v2.1 [41]. Volumes of interest for source organs (kidneys, spleen, liver, total body, heart contents, and lumbar vertebrae) were either manually drawn or corrected if automatic contouring tools were initially used. VOIs were defined by manual delineation. Central image review was conducted by 1 central nuclear medicine physician with previous [^89^Zr]Zr-girentuximab PET interpretation experience. Tumor uptake was assessed by analyzing [^89^Zr]Zr-girentuximab accumulation in tumors or surrounding regions according to the whole-body PET images and compared with the pre-screening CT or MRI images. Lesions of interest were considered PET positive or PET negative based on a visual scale, taking into account the background signal in surrounding organs and blood pool. PET positivity was defined as increased uptake of a mass compared to surrounding tissue. Tumor dosimetry analysis was performed on lesions showing [^89^Zr]Zr-girentuximab uptake on PET using the spherical model and voxel S method [42]. For specific tumor masses, the dose was calculated by interpolation using a power function to the previously determined volume of the lesion on the pre-screening CT or MRI images, assuming a density of 1.06 g/cm^3^.

### Statistical analysis

Radiation exposure, laboratory tests, and vital signs were summarized using descriptive statistics.

Radiation dosimetry was analyzed using OLINDA/EXM v2.2 and IDAC-Dose v2.1 software. Output of OLINDA/EXM and IDAC-Dose were absorbed dose, normalized absorbed dose, whole-body effective dose, and whole-body normalized effective dose. Organ doses in OLINDA/EXM were reported in Sv and refer to equivalent doses; absorbed doses were reported in units of Gy. Descriptive statistics were used, and data were expressed as either percentages or medians (with ranges). The Mann-Whitney U test was used to compare the distributions of tumor normalized absorbed doses for patients with and without confirmed ccRCC.

Whole blood PK parameters were calculated by non-compartmental analysis based on the actual sampling time relative to the start of administration using Phoenix WinNonlin 8.3. Parameters included area under the concentration-time curve to last time point (AUC_0−t_), area under the concentration-time curve extrapolated to infinity (AUC_0−∞_), maximum serum concentration (C_max_), time to C_max_ (T_max_), estimate of the terminal elimination rate constant (λ_z_), and elimination half-life. Details on pharmacokinetic parameter methodology can be found in Supplementary Table 1.

Statistical analyses were performed using SAS software, version 9.4 (SAS Institute Inc., Cary, NC, USA).

## Results

### Patient characteristics

Twelve Chinese patients were screened for eligibility. Of these, 2 withdrew consent voluntarily during screening; 10 patients enrolled and completed the study. Mean (SD) age was 57.2 (15.7) years; 6 (60%) were male (Table 1). All patients had a clinical diagnosis of IRMs, suspected renal cell carcinoma.

**Table 1:** Demographics and baseline characteristics

Characteristics	N = 10
Mean age (SD)^a^	57.2 years (15.7)
Male sex, n (%)	6 (60.0)
Female sex, n (%)	4 (40.0)
Body Mass Index (kg/m^2^)	
Mean (SD)	24.89 (2.38)
Median (range)	24.72 (21.3–30.0)
Glomerular filtration rate (mL/min/1.73m^2^)	
Mean (SD)	99.8 (13.0)
Median (range)	100.4 (82.6–120.6.6.6)
Clinical diagnosis, n (%)	
Indeterminate renal mass, suspected renal cell carcinoma	10 (100)
Suspected recurrent clear cell renal cell carcinoma	0

^a^Age is calculated based on age at time of informed consent

All patients had 1 renal tumor, and 1 patient also had extrarenal tumors in lymph node and lung visualized by enhanced CT. Renal tumor size ranged from 18 mm to 119 mm, and extrarenal tumor size ranged from 15 mm to 29 mm. Seven patients had histologically confirmed ccRCC. The other 3 patients had histologically confirmed TFE3 translocation RCC, chromophobe RCC, and RCC (excluding RCC with Mit family translocation). All 10 patients were included in safety, dosimetry, and PK analyses.

## Safety

The mean (SD) decay corrected activity administered was 36.43 (0.68) MBq, with a range from 35.52 to 37.76 MBq. Of 10 AEs reported in 8 patients (Table 2), 9 (90%) AEs were Grade 1 or 2. One patient experienced 1 event of Grade 3 anemia; this patient had a medical history of Grade 2 anemia at the screening visit. No AEs were considered related to [^89^Zr]Zr-girentuximab, and no serious adverse events were reported.

**Table 2:** Adverse events

System organ class and preferred term (MedDRA 26.0)	Grade 1	Grade 2	Grade 3	Total
Investigations, n (%)	4 (40.0)	2 (20.0)	0	6 (60.0)
Blood glucose increased	2 (20.0)	0	0	2 (20.0)
Urinary occult blood positive	2 (20.0)	0	0	2 (20.0)
White blood cells urine positive	2 (20.0)	0	0	2 (20.0)
Bilirubin conjugated increased	0	1 (10.0)	0	1 (10.0)
Blood pressure increased	0	1 (10.0)	0	1 (10.0)
Glomerular filtration rate decreased	1 (10.0)	0	0	1 (10.0)
Glucose urine present	1 (10.0)	0	0	1 (10.0)
Neutrophil count decreased	1 (10.0)	0	0	1 (10.0)
Protein total decreased	1 (10.0)	0	0	1 (10.0)
Red blood cells urine positive	1 (10.0)	0	0	1 (10.0)
White blood cell count decreased	1 (10.0)	0	0	1 (10.0)
Blood and lymphatic system disorders, n (%)	2 (20.0)	0	1 (10.0)	3 (30.0)
Anemia	2 (20.0)	0	1 (10.0)	3 (30.0)
Cardiac disorders, n (%)	2 (20.0)	0	0	2 (20.0)
Atrioventricular block first degree	2 (20.0)	0	0	2 (20.0)
Injury, poisoning and procedural complications, n (%)	2 (20.0)	0	0	2 (20.0)
Post procedural inflammation	2 (20.0)	0	0	2 (20.0)
Metabolism and nutrition disorders, n (%)	1 (10.0)	1 (10.0)	0	2 (20.0)
Hypoalbuminemia	1 (10.0)	0	0	1 (10.0)
Hypocalcaemia	0	1 (10.0)	0	1 (10.0)
Hyponatremia	1 (10.0)	0	0	1 (10.0)
Gastrointestinal disorders, n (%)	0	1 (10.0)	0	1 (10.0)
Vomiting	0	1 (10.0)	0	1 (10.0)

The severity of adverse events was graded according to National Cancer Institute-Common Toxicity Criteria for Adverse Events version 5.0. Patients with multiple occurrences of adverse events in the same preferred term or system organ class are counted only once within that preferred term or system organ class. Percentages are based on the number of all included patients (*N* = 10). No adverse events were considered to be related to [89Zr]Zr-girentuximab

All patients had a negative human anti-chimeric Ab (HACA) result at baseline. One patient (10%) showed a positive result at the final visit, which was 15 days after [^89^Zr]Zr-girentuximab administration. No AEs were observed for this patient.

## Organ radiation dosimetry

The organs that received the highest normalized absorbed doses (mean [SD]) according to OLINDA/EXM v2.2 were the liver (1.51 [0.23] mGy/MBq), kidneys (1.42 [0.35] mGy/MBq), heart wall (1.22 [0.18] mGy/MBq), adrenals (0.97 [0.15] mGy/MBq), and spleen (0.93 [0.51] mGy/MBq) (Table 3). Normalized absorbed doses were also calculated according to IDAC-Dose v2.1 (Supplementary Table 2).

**Table 3:** Normalized absorbed dose to organs calculated using OLINDA/EXM v2.2 (mGy/MBq)

Organ	N = 10 Mean mGy/MBq (SD)
Liver	1.51 (0.23)
Kidneys	1.42 (0.35)
Heart wall	1.22 (0.18)
Adrenals	0.97 (0.15)
Spleen	0.93 (0.51)
Gallbladder wall	0.79 (0.08)
Pancreas	0.66 (0.09)
Red marrow	0.62 (0.15)
Stomach wall	0.58 (0.05)
Esophagus	0.56 (0.07)
Left colon	0.55 (0.09)
Thymus	0.54 (0.05)
Right colon	0.53 (0.03)
Osteogenic cells	0.51 (0.8)
Ovaries	0.50 (0.01)
Lungs	0.50 (0.06)
Small intestine	0.48 (0.03)
Uterus (*n* = 4)	0.48 (0.01)
Rectum	0.44 (0.05)
Total body	0.40 (0.6)
Prostate	0.39 (0.02)
Urinary bladder wall	0.37 (0.02)
Thyroid	0.37 (0.02)
Breasts	0.37 (0.01)
Salivary glands	0.34 (0.02)
Testes (*n* = 6)	0.30 (0.02)
Eyes	0.29 (0.03)
Brain	0.29 (0.03)
Whole-body normalized effective dose (mSv/MBq)	0.49 (0.08)

The mean (SD) whole-body normalized effective dose following administration of [^89^Zr]Zr-girentuximab PET was 0.49 (0.08) mSv/MBq using OLINDA/EXM v2.2.

### Tumor dosimetry

Tumor dosimetry was performed in all 10 patients (9 renal tumors and 1 external lymph node distant metastasis). Two tumors were not evaluable in the 1 patient with 3 tumors; the absorbed dose of a renal tumor and extrarenal lung distant metastasis could not be estimated due to limitations of the spherical model because the size of the renal tumor was > 1000 g and the extrarenal tumor was < 1 g. The mean (SD) tumor normalized absorbed dose was 2.19 (1.85) mGy/MBq (range: 0.12 to 5.41 mGy/MBq) for the renal tumors and 1.17 mGy/MBq for the extrarenal tumor. In the 7 patients with histologically confirmed ccRCC, mean (SD) tumor normalized absorbed dose was 2.87 (1.54) mGy/MBq (range: 1.17 to 5.41 mGy/MBq) and median (interquartile range) tumor normalized absorbed dose was 2.85 [1.28 to 3.88] mGy/MBq. In the 3 patients with non-ccRCC renal lesions, median (interquartile range) tumor normalized absorbed dose was significantly lower (0.31 [0.12–0.42] mGy/MBq; *p* = 0.023).

### [^89^Zr]Zr-girentuximab PET imaging

**Fig. 1:**
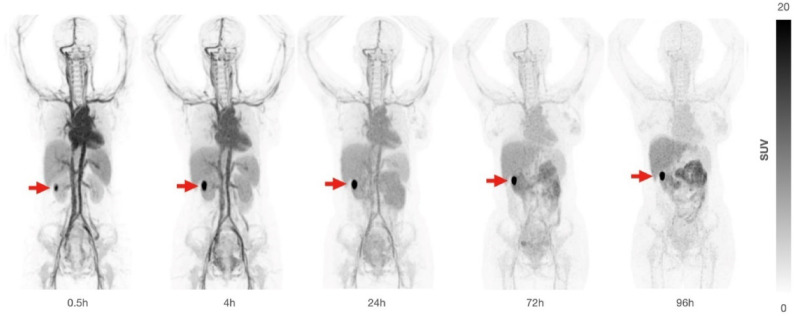
Whole-body maximum-intensity projections over time after [^89^Zr]Zr-girentuximab administration. Mass dose of 10 mg girentuximab demonstrating a ccRCC tumor in right kidney (red arrow) at 0.5 hours with an increased tumor to background ratio over time. Standardized uptake values were normalized to body weight

All 7 patients with histologically confirmed ccRCC had positive PET imaging results. Lesions were visible on PET imaging in 5 patients at 4 h and in 6 patients at 24 h after [^89^Zr]Zr-girentuximab administration; in 1 patient, tumor lesion was already visible at 0.5 h (Fig. 1). In the 3 patients with non-ccRCC renal lesions, 2 (chromophobe RCC and RCC [excluding RCC with Mit family translocation]) had negative PET imaging results and 1 (TFE3 translocation RCC) had equivocal PET imaging result.

### Pharmacokinetics

**Fig. 2:**
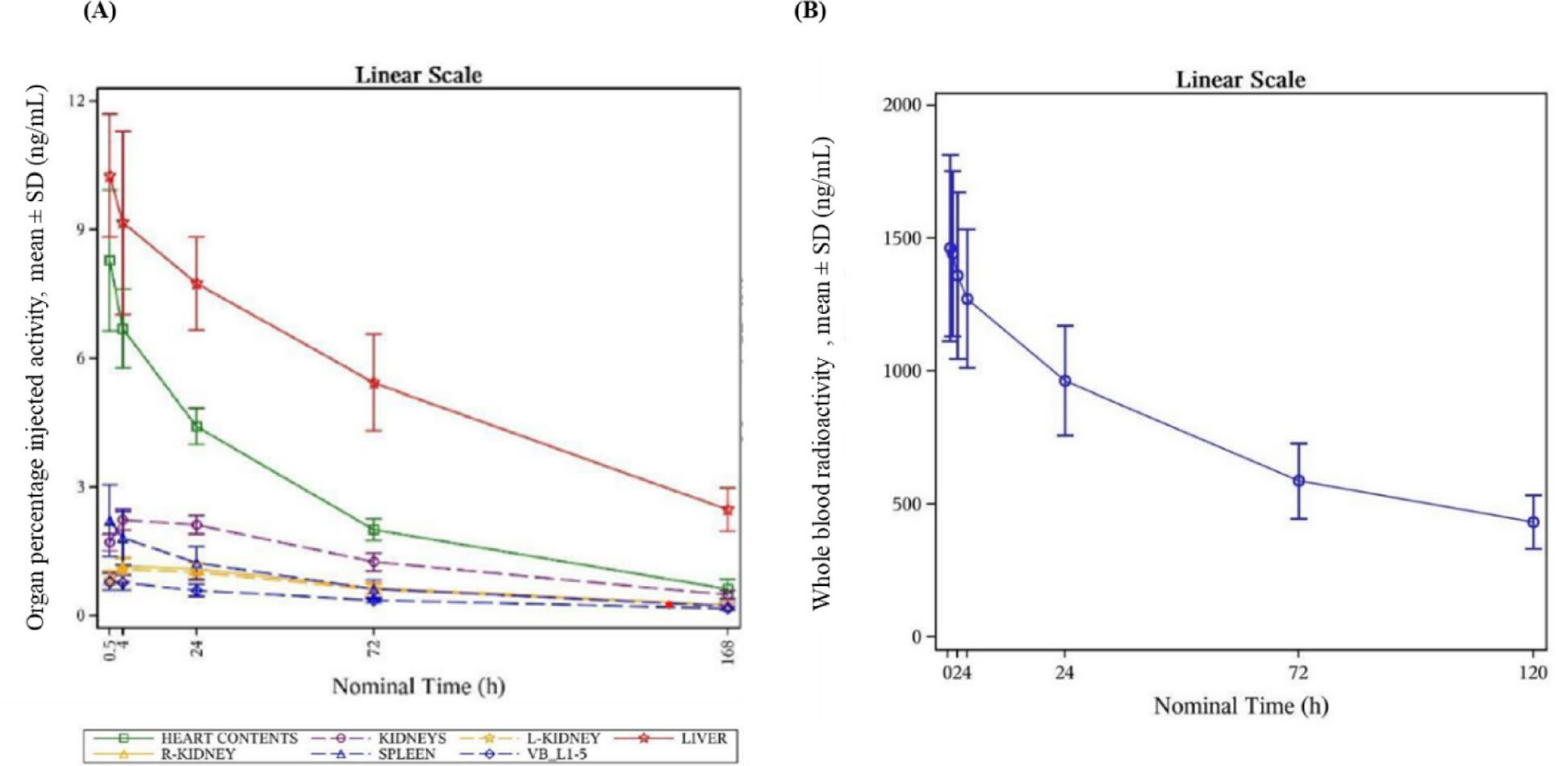
(**A**) Mean (SD) organ percentage injected activity-time plot and (**B**) mean (SD) whole blood concentration-time plot of [^89^Zr]Zr-girentuximab radioactivity

Mean (SD) time to maximum concentration of whole blood radioactivity of [^89^Zr]Zr-girentuximab was approximately 0.85 (0.25) hour (range: 0.53 to 1.10 h) after administration and declined slowly with a mean (SD) elimination half-life of 78.73 (11.90) hours (range: 64.13 to 95.68 h) **(**Fig. 2B**)**. Trough concentration was reached on Day 5. Mean (SD) C_max_ of whole blood radioactivity of [^89^Zr]Zr-girentuximab was 1478.20 (333.56) ng/mL, and mean (SD) AUC_0−inf_ estimated over the period from injection to infinity was 139095.44 (41816.24) ng*h/mL. Mean (SD) total body clearance rate was 73.83 (21.33) mL/hour with an estimated mean (SD) volume of distribution of 8109.13 (1452.14) mL.

## Discussion

To our knowledge, this study is the first to report safety and tolerability and dosimetry of [^89^Zr]Zr-girentuximab in Chinese patients. Results are in line with previously reported studies conducted in other populations [30, 31]. Our results suggest potential applicability of other relevant study results, such as from the ZIRCON study demonstrating high diagnostic performance for detection and characterization of ccRCC [32], to Chinese patients. In this study, 5 (71%) of the 7 patients with histologically confirmed ccRCC had tumor lesions visible on a total-body scanner at 4 h after [^89^Zr]Zr-girentuximab administration; additional studies are needed to confirm the value of early-phase imaging with [^89^Zr]Zr-girentuximab PET.

Girentuximab is a mAb with a longer uptake and clearance time compared with small molecules. Radiolabeling with [^89^Zr]Zr is advantageous for imaging with mAbs due to its long physical half-life of 78.4 h [32]. The long half-life allows for dose optimization sufficient for clear images with high tumor-to-background ratios and standardized uptake values. Our findings provide preliminary evidence that [^89^Zr]Zr-girentuximab PET may help differentiate ccRCC from other renal lesions in Chinese patients, although confirmation in larger cohorts is warranted. Consistent with previous studies, we observed lower tumor uptake of [^89^Zr]Zr-girentuximab in non-ccRCC versus ccRCC lesions [30, 32]. While a negative [^89^Zr]Zr-girentuximab PET finding may suggest a benign mass or indolent RCC, confirmatory imaging is recommended to establish a definitive diagnosis. Notably, non-ccRCC subtypes that express CAIX are often aggressive malignancies [43, 44]; for example, CAIX-positive papillary RCC has been associated with higher stage, higher grade, and poorer prognosis [45]. Therefore, detection of a CAIX-avid lesion on [^89^Zr]Zr-girentuximab PET may provide clinically relevant information even in non-ccRCC. Of interest, we report 1 patient with an extrarenal lesion detected on CT and [^89^Zr]Zr-girentuximab PET, suggesting feasibility of [^89^Zr]Zr-girentuximab for detection of extrarenal lesions. The role of CAIX-targeting for metastatic lesion detection has not been fully elucidated and warrants further investigation, especially given potential of targeted theranostics for patients with CAIX-expressing solid tumors [30, 33].

Similar to previous [^89^Zr]Zr-girentuximab studies [30–32], most AEs were mild in intensity. The most common AE was anemia (3 [30%] patients). No new safety signals were observed. While 1 patient showed a positive HACA result 15 days after administration, no AEs were observed. Additional studies are needed to determine the relevance of HACA with [^89^Zr]Zr-girentuximab after repeated administrations. Additional safety data of [^89^Zr]Zr-girentuximab in Chinese patients will be collected as part of the Phase 3 ZIRCON-CP study (NCT06750419).

[^89^Zr]Zr-girentuximab accumulated more slowly in tumors compared with normal tissue, and dosimetry analysis showed that the organs receiving the highest doses were the liver and the kidneys. The biodistribution and dosimetry of [^89^Zr]Zr-girentuximab in Chinese patients was comparable to previous studies conducted in European and Japanese populations [30, 31]. The renal tumor normalized absorbed dose range (0.12 to 5.41 mGy/MBq) overlapped with the ranges (1.90 to 11.6 mGy/MBq, and 0.67 to 6.55 mGy/MBq) reported in previously published studies calculated on OLINDA/EXM 2.1 and OLINDA, respectively [30, 31].

This study has several limitations. First, the overall sample size was small, and in particular, the number of non-ccRCC lesions was limited. Second, only 1 patient in our study had documented metastasis, which precludes broader generalization of findings in the metastatic setting. Third, normal organ and tumor segmentation was performed by a single reader, which may introduce bias. Finally, imaging was conducted on a total-body PET scanner, enabling rapid acquisition (10 min for whole-body imaging). The duration for whole-body imaging may vary depending on PET technology used.

Despite these limitations, this study extends prior findings from the Phase 3 ZIRCON study by providing preliminary evidence in a Chinese patient population; the diagnostic utility of [^89^Zr]Zr-girentuximab will be further assessed in the Phase 3 ZIRCON-CP study. Overall, results contribute to the understanding of dosimetry, safety, and pharmacokinetics in Chinese patients, thereby addressing current evidence gaps and supporting broader generalizability of [^89^Zr]Zr-girentuximab PET across diverse patient groups.

## Conclusion

[^89^Zr]Zr-girentuximab has a favorable safety and tolerability profile in Chinese patients, with a similar organ radiation and tumor dosimetry as previously reported in other populations [30, 31]. Our results support existing data that suggest that [^89^Zr]Zr-girentuximab is a promising non-invasive PET agent for detection and characterization of ccRCC. CAIX-targeted radiotracers, including [^89^Zr]Zr-girentuximab, are being actively investigated and may play an important role in future theranostic strategies for ccRCC.

## Supplementary Information

Supplementary Material 1

Supplementary Material 2

## Data Availability

The de-identified patient dataset pertaining to results reported in this manuscript will be made available upon reasonable request to the corresponding author after the intervention and indication is approved by both the US Food and Drug Administration, European Medicines Agency, and Chinese National Medical Products Administration.

## Abbreviations

AE: Adverse event
AUC: Area under the curve
CAIX: Carbonic anhydrase IX
ccRCC: Clear cell renal cell carcinoma
CT: Computed tomography
GCP: Good Clinical Practice
GPP: Good Publication Practice
HACA: Human anti-chimeric Ab
mAbs: Monoclonal antibodies
MRI: Magnetic resonance imaging
IRM: Indeterminate renal mass
PET: Positron emission tomography
PK: Pharmacokinetic
SD: Standard deviation
