# Supplementary material for: Evaluation of [89Zr]Zr-girentuximab PET imaging of clear cell renal cell carcinoma in Chinese patients: a Phase 1 clinical study (ZIRDOSE-CP)
Source: EJNMMI Res. 2025 Dec 24;16:1. doi: 10.1186/s13550-025-01332-5 (PMC12764708; doi:10.1186/s13550-025-01332-5)
Supplement: Supplementary file 2 — Supplementary Material 2 [file 13550_2025_1332_MOESM2_ESM.docx]

Supplemental Information

**Pharmacokinetic Parameters**

Supplementary Table 1. Derivation of Pharmacokinetic Parameters

| **Parameter** | **Definition** | **Method of Determination** |
| --- | --- | --- |
| AUC_0-t_ | Area under the concentration-time curve from 0 to t hours | Linear up to t_max_, log-linear from t_max_ down to t_last_ |
| AUC_0-inf_ | Area under the concentration-time curve from 0 to infinity | AUC_0-t_ + C_last_/λ_z_, where C_last_ is the calculated whole blood concentration at the last quantifiable time point, and λ_z_ estimated from the log-linear regression analysis |
| C_max_ | Maximum concentration | Observed directly from data |
| t_max_ | Time to maximum concentration | Observed directly from data as time of first occurrence |
| t_1/2_ | Elimination half-life | 0.693/λ_z_, only those data points judged to describe the terminal log-linear decline will be used in the regression |
| λ_z_ | Terminal elimination rate constant | Calculated by a linear regression through at least 3 data points in the terminal phase of the log-linear concentration-time curve |
| CL_t_ | Total body clearance | Dose/AUC_0-inf_ |
| Vd_z_ | Volume of distribution | Dose/( AUC_0-inf_*λ_z_) |

**Organs Receiving Highest Normalized Doses**

The organs that received the highest normalized absorbed doses (mean [SD]) according to IDAC-Dose v2.1 were the kidneys (1.36 [0.26] mGy/MBq), liver (1.30 [0.21] mGy/MBq), gallbladder wall (0.95 [0.10] mGy/MBq), adrenals (0.86 [0.09] mGy/MBq), and spleen (0.79 [0.11] mGy/MBq).

Supplementary Table 2. Normalized Absorbed Dose to Organs Calculated Using IDAC-Dose v2.1 (mGy/MBq)

| **Organ** | **N = 10**  **Mean mGy/MBq (SD)** |
| --- | --- |
| Kidneys | 1.36 (0.26) |
| Liver | 1.30 (0.21) |
| Gallbladder wall | 0.95 (0.10) |
| Adrenals | 0.86 (0.09) |
| Spleen | 0.79 (0.11) |
| Pancreas | 0.67 (0.05) |
| Red marrow | 0.58 (0.12) |
| Ureters | 0.55 (0.02) |
| Stomach wall | 0.53 (0.06) |
| Ovaries (n = 4) | 0.50 (0.01) |
| Uterus/cervix (n = 4) | 0.49 (0.01) |
| Oesophagus wall | 0.47 (0.04) |
| Heart wall | 0.47 (0.03) |
| Right colon wall | 0.46 (0.02) |
| Si wall | 0.45 (0.05) |
| Systemic lymph nodes | 0.45 (0.04) |
| Lymphatic nodes | 0.44 (0.04) |
| Colon | 0.43 (0.02) |
| Bronchial bound region | 0.41 (0.04) |
| Alveolar-interstitium | 0.41 (0.03) |
| Bronchiolar sequestered region | 0.41 (0.03) |
| Lungs | 0.41 (0.03) |
| Thoracic lymph nodes | 0.41 (0.03) |
| Prostate (n = 6) | 0.41 (0.02) |
| Bronchial sequestered region | 0.40 (0.04) |
| Left colon wall | 0.40 (0.02) |
| Rectosigmoid colon wall | 0.39 (0.04) |
| Urinary bladder wall | 0.39 (0.02) |
| Extrathoracic lymph nodes | 0.38 (0.05) |
| Thymus | 0.38 (0.05) |
| Cortical bone mineral surface | 0.37 (0.06) |
| Muscle | 0.36 (0.04) |
| Thyroid | 0.36 (0.04) |
| Pituitary gland | 0.35 (0.05) |
| Tonsils | 0.35 (0.03) |
| Adipose | 0.35 (0.02) |
| Breasts | 0.34 (0.03) |
| Oral mucosa | 0.32 (0.03) |
| Testes (n = 6) | 0.32 (0.02) |
| Salivary glands | 0.31 (0.04) |
| Brain | 0.31 (0.03) |
| Et region | 0.29 (0.04) |
| Et2 surface | 0.29 (0.04) |
| Tongue | 0.27 (0.02) |
| Skin | 0.25 (0.02) |
| Eye lenses | 0.21 (0.02) |
| Et1 surface | 0.19 (0.04) |
| **Whole-body normalized effective dose (mSv/MBq)** | **0.50 (0.02)** |
