# Supplementary material for: Evaluation of [89Zr]Zr-girentuximab PET imaging of clear cell renal cell carcinoma in Chinese patients: a Phase 1 clinical study (ZIRDOSE-CP)
Source: EJNMMI Res. 2025 Dec 24;16:1. doi: 10.1186/s13550-025-01332-5 (PMC12764708; doi:10.1186/s13550-025-01332-5)
Supplement: Supplementary file 1 — Supplementary Material 1 [file 13550_2025_1332_MOESM1_ESM.docx]

Dr Irene A Burger

Editor-in-Chief

*EJNMMI Research*

25 September 2025

RE: Manuscript Number EJRE-D-25-00217R1

Title: Evaluation of ^89^Zr-girentuximab PET imaging of clear cell renal cell carcinoma in Chinese patients: A Phase 1 clinical study (ZIRDOSE-CP)

Dear Dr Burger,

Thank you for providing reviewer comments for our manuscript.

We have revised the manuscript to address reviewer comments. Our point-by-point responses are provided below, and changes within the manuscript are shown with tracked changes. A clean file is also provided.

We hope you and the editor find these revisions suitable and that the manuscript is acceptable for publication in *EJNMMI Research*.

Sincerely,

David Cade

**Reviewer #1 comments:**

This Phase 1 study assessed the safety, dosimetry, and pharmacokinetics of ⁸⁹Zr-girentuximab PET in Chinese patients with suspected clear cell renal cell carcinoma. Results indicated good safety, accurate tumor targeting, and radiation dosimetry similar to non-Chinese populations, supporting its potential as a noninvasive diagnostic tool for ccRCC. Below are minor but important revisions to improve clarity, reproducibility, and compliance with nuclear medicine standards:

These minor revisions will improve the manuscript's transparency, reproducibility, and alignment with best practices in nuclear medicine.

1. Clearly specify the radiochemical purity threshold (e.g., >95%) for the administered ⁸⁹Zr-girentuximab and the QC methods used (e.g., iTLC, HPLC).

**Response:** We have added the following statement to the Methods:

*“Radiochemical purity of [^89^Zr]Zr-girentuximab was confirmed to be ≥90% by both instant thin-layer chromatography and size-exclusion high-performance liquid chromatography.”*

2. State whether radiolabeling was conducted in-house or supplied by an external GMP facility, and briefly describe the labeling method or cite a previously validated procedure.

**Response:** We have added the following statements to the Methods:

*“Radiolabeling was conducted at JFE Engineering, a GMP-certified Contract Manufacturing Organization. The radiolabeling process involved conjugation of girentuximab with the chelator desferrioxamine-tetrafluorophenol (DFO-TFP) to produce the intermediate ^89^Zr-DFO-girentuximab. The study dose consisted of 37 MBq of ^89^Zr conjugated to girentuximab, diluted with unconjugated girentuximab in 0.9% sodium chloride to achieve a total antibody dose of 10 mg/10 mL for intravenous administration.”*

3. Specify the PET/CT scanner model, acquisition mode (e.g., 3D list-mode), reconstruction algorithm (e.g., OSEM with specific iterations/subsets), and matrix size. Clarify the low-dose CT protocol (e.g., kVp, mAs, pitch) used for attenuation correction.

**Response:** We have added the following statements to the Methods:

*“All PET/CT scans were performed on a UEXPLORER (serial number F00014; United Imaging, Shanghai, China). PET data were acquired in 3D list-mode and reconstructed using an ordered-subset expectation maximization algorithm (3 iterations, 20 subsets; matrix size 192 × 192; pixel size 3.125 × 3.125 mm; slice thickness 2.886 mm, no overlap). PET acquisition duration was 10 minutes for one bed position on the total-body scanner. Low-dose CT was acquired at 120 kVp with a spiral pitch factor of 1.0125, revolution time 0.5 s, and a collimation width of 0.5 mm (total collimation 40 mm). CT images were reconstructed with a slice thickness of 3 mm (50% overlap) using a 512 × 512 matrix (pixel size 0.977 × 0.977 mm). Standard quantitative corrections for normalization, randoms, dead time, decay, attenuation, and scatter, and time-of-flight and point spread function modeling were applied to all PET/CT imaging data.”*

4. Include voxel size, criteria for ROI definition (e.g., fixed threshold, %SUVmax), and indicate whether segmentation was manual or semi-automated.

**Response:** [^89^Zr]Zr-girentuximab PET scan interpretation is based on visual examination only. In addition to the statements added in response to comment 3, we have added the following statement to the Methods:

*“Volumes of interest (VOIs) for source organs (kidneys, spleen, liver, total body, heart contents, and lumbar vertebrae) were either manually drawn or corrected if automatic contouring tools were initially used. VOIs were defined by manual delineation.”*

5. Use consistent tracer nomenclature: follow SNMMI/EANM guidelines for [^89^Zr]Zr-girentuximab (or 89Zr-girentuximab if the journal prefers simplified formatting), maintaining consistency.

**Response:** We have changed the nomenclature used throughout manuscript from “^89^Zr-girentuximab” to “[^89^Zr]Zr-girentuximab” as suggested.

6. Briefly discuss the implications of low tumor uptake in non-ccRCC subtypes, including the potential impact on negative predictive value in clinical decision-making.

**Response:** We have added the following statements to the discussion section to address the implications of low tumor uptake in non-ccRCC subtypes:

*“Consistent with previous studies, we observed lower tumor uptake of [^89^Zr]Zr-girentuximab in non-ccRCC versus ccRCC lesions (30,32). While a negative [^89^Zr]Zr-girentuximab PET finding may suggest a benign mass or indolent RCC, confirmatory imaging is recommended to establish a definitive diagnosis. Notably, non-ccRCC subtypes that express CAIX are often aggressive malignancies (43,44); for example, CAIX-positive papillary RCC has been associated with higher stage, higher grade, and poorer prognosis (45). Therefore, detection of a CAIX-avid lesion on [^89^Zr]Zr-girentuximab PET may provide clinically relevant information even in non-ccRCC.”*

**Reviewer #2 comments:**

Although the study presents novel and clinically relevant data on [^89Zr]Zr-girentuximab PET/CT imaging in Chinese patients with suspected ccRCC, and the dataset itself appears robust and well-documented, the manuscript still falls short in a number of key areas:

First, the nomenclature of the radiopharmaceutical remains inconsistent throughout the manuscript, figure legends, and even within the response letter. The format "[^89Zr]Zr-girentuximab," as per IUPAC and EJNMMI standards, must be strictly adhered to in all sections. This lack of standardization is not a trivial issue, as it can affect clarity, scientific accuracy, and compliance with journal requirements.

**Response:** We have changed the nomenclature used throughout manuscript from “^89^Zr-girentuximab” to “[^89^Zr]Zr-girentuximab” as suggested.

Second, the transparency and methodological rigor surrounding image analysis and presentation have not been sufficiently improved. The figures and their legends do not specify basic yet essential details such as the number of patients represented, the normalization method for SUV, the anonymization procedures for patient data, or the type and source of concentration units in PK curves. These omissions not only hinder reproducibility but also raise potential concerns about compliance with international standards for data presentation and research ethics.

**Response:** We have added methodological details to the Methods and revised figure legends. Details on all revisions are noted in table below.

Third, although the authors responded to editorial concerns about objectivity and potential bias, the revised discussion still lacks a clear demonstration of a more critical, balanced, and less promotional tone. The changes are described only in general terms, and the manuscript would benefit from explicit language acknowledging the study's limitations, potential sources of bias, and the distinction between current data and commercial aspirations for the tracer.

**Response:** We appreciate the reviewer’s comment. We have added statements on limitations to the Discussion.

*“This study has several limitations. First, the overall sample size was small, and in particular, the number of non-ccRCC lesions was limited. Second, only 1 patient in our study had documented metastasis, which precludes broader generalization of findings in the metastatic setting. Third, normal organ and tumor segmentation was performed by a single reader, which may introduce bias. Finally, imaging was conducted on a total-body PET scanner, enabling rapid acquisition (10 minutes for whole-body imaging). The duration for whole-body imaging may vary depending on PET technology used.”*

Fourth, the manuscript does not fully address reviewer queries about adverse events, in particular the attribution of anemia cases. The response relies primarily on temporal association and patient history, rather than providing clear, objective criteria or reference to accepted methods for adverse event causality assessment.

**Response:** We thank the reviewer for this important comment. In our study, adverse events (AEs) were defined as any event that newly occurred or worsened after administration of [^89^Zr]Zr-girentuximab. Attribution of AEs was determined by the investigators using predefined criteria, consistent with the published Phase 3 ZIRCON study (Shuch B, et al. *Lancet Oncol*. 2024;25(10):1277-1287.).

We have added the following detail to the Methods to address adverse event assessments.

***“****The severity of AEs was graded according to National Cancer Institute-Common Toxicity Criteria for Adverse Events version 5.0.****”***

In addition, I have attached a file listing the specific issues identified in this revision. My greatest concern centers around the relatively serious methodological and reproducibility errors, which could have a significant impact on the reliability and scientific value of the manuscript. Ensuring full transparency and methodological clarity is essential for a study of this nature and scope.

**Response:** Thank you for providing a detailed list of specific issues to address in our manuscript. The table below outlines the revisions we implemented to the manuscript to address your comments.

| **Section** | **Reviewer Comment** | **Response** |
| --- | --- | --- |
| **Abstract** | Absence of the Methods subtitle, causing a confusing and inadequate structure for the abstract. | We thank the reviewer for this suggestion. However, per *EJNMMI Research* journal guidelines, abstracts are not structured with a “Methods” subtitle. We have ensured that the abstract text describes the methods while adhering to journal formatting requirements. |
|  | Absence of mention of ethical approval/ethics committee and informed consent. | Ethics approval and informed consent are now explicitly mentioned in abstract.  *“This study was approved by the Ethics Committee of Beijing Cancer Hospital (2022YW225) and was conducted in accordance with the Declaration of Helsinki. All patients provided written informed consent.”* |
|  | The standard is: Trial registration: ClinicalTrials.gov Identifier: NCT05861778. Registered 17 May 2023. The hyperlink is unnecessary or should be in parentheses. | Trial registration now presented as: Trial registration ClinicalTrials.gov Identifier: NCT05861778. Registered 17 May 2023. |
| **Keywords** | The journal requires the keywords to be separated by a semicolon (;) and all on the same line, without bullets or markers. | Keywords have been reformatted according to journal requirements. |
|  | 89 Zr-girentuximab” is incorrect; it should be [^89Zr]Zr-girentuximab | We have changed the nomenclature used throughout manuscript from “^89^Zr-girentuximab” to “[^89^Zr]Zr-girentuximab”. |
|  | “CAIX” alone is not a MeSH term; the correct term is “Carbonic Anhydrase IX” | We have corrected the keyword to Carbonic Anhydrase IX. |
| **Background** | “While incidence of ccRCC has increased (8), technological advancements to improve early detection and appropriate treatment selection have not advanced at the same rate” – Error: potentially subjective statement with no concrete data and no explanation for the increase in incidence | We have revised the statement to include concrete data and explanation for the increased incidence:  *“While the incidence of ccRCC is increasing globally (3.9 per 100,000 in 1990 to 4.5 per 100,000 in 2021) (1) due to factors such as improved imaging techniques and lifestyle changes (8, 9), technological advancements in early detection and treatment selection remain limited (10-19).”* |
|  | “while specificity may be as low as 33%” – Continuity error: is this value for CT or MRI? | We have revised the statement for clarity:  *“However, the sensitivity for predicting ccRCC in patients with suspicious small renal masses of both CT and MRI are moderate (79.7% and 88.1%, respectively), while specificity may be as low as 44% for CT and 33% for MRI”* |
|  | case reports showcasing real-world experience also demonstrate clinical utility and clinical decision-making impact of 89 Zr-girentuximab – Continuity/clarity error: what was the utility? What was the impact at the time of decision-making? | We have revised the statement for clarity:  *“The clinical decision-making impact of [^89^Zr]Zr-girentuximab PET has also been demonstrated in published patient case reports including from real-world experience, demonstrating its utility for guiding surgical planning, providing confidence in treatment plan, and avoiding unnecessary interventions”* |
| **Materials & Methods – Patients and Procedure** | The text mentions informed consent, but does not state whether the study was approved by an ethics committee (name, number, country). This is mandatory in clinical research articles. | Ethics information was previously noted in the Declarations section. We have moved this information to the main text. We have also added the ethics committee application number (2022YW225). Please note that there is no ethics committee number in China. |
|  | “glomerular filtration rate ≤60 mL/min/1.73 m²” – Continuity/clarity error: was this at the time of diagnosis or before administration of the radiopharmaceutical? | We have clarified the statement by adding timing:  *“renal insufficiency with glomerular filtration rate ≤60 mL/min/1.73 m^2^ within 30 days before planned [^89^Zr]Zr-girentuximab administration”* |
| **Assessments** | “Adverse events (AEs) were recorded from time of 9 Zr-girentuximab...” – Typo: the 8 before the 9 is missing. | We have corrected this typo. |
|  | “if: (a) the event is more likely to be caused by the patient's clinical status or other treatment methods…” — Methodological error: vague criterion, with no reference to an international standard (e.g., CTCAE). | We have added the following statement to the Methods:  *“The severity of AEs was graded according to National Cancer Institute-Common Toxicity Criteria for Adverse Events version 5.0.”* |
|  | “spherical model and voxel S method” — Reproducibility error: not explained or referenced | We have added references to the methods section for reproducibility. |
| **Statistical Analysis** | “Organ doses in OLINDA/EXM were reported in Sv and refer to equivalent doses instead of absorbed doses, which were reported in units of Gy. In this study with 89 Zr, the two units were assumed identical.” – Clarity/continuity error: Gy (gray) is a unit of absorbed dose; Sv (sievert) is a unit of equivalent/effective dose. They are not identical and cannot be assumed as such unless (explicitly) weighting factor = 1, which must be detailed and justified with a reference. | We agree with your definition and have revised the units used throughout the manuscript for accuracy. |
|  | “The Mann-Whitney U test was used to compare mean tumor normalized absorbed doses.” – Conceptual error: The Mann-Whitney U test compares medians, not means; the correct is to report that it compares distributions or medians between groups. | We have revised the statement to the following for accuracy:  *“The Mann-Whitney U test was used to compare the distributions of tumor normalized absorbed doses for patients with and without confirmed ccRCC.”* |
|  | If possible, add the description of general statistical software and whether the tests are one-sided/two-sided or the significance value adopted. | We have added the following statement to the Methods:  *“Statistical analyses were performed using SAS software, version 9.4 (SAS Institute Inc., Cary, NC, USA).”* |
| **Results** | “Seven patients had histologically confirmed ccRCC. The other 3...” – Ambiguity error: it is ambiguous whether the 3 non-ccRCC patients were included in the efficacy analyses (dosimetry, response, etc.). | We have added a statement to clarify the analysis populations:  *“All 10 patients were included in safety, dosimetry, and PK analyses.”* |
| **Safety** | It is not stated which criterion/standard was used to classify AEs as “Grade 1”, “Grade 2”, “Grade 3” (e.g., CTCAE, MedDRA, etc.). | We have added the following statement to the Methods:  *“The severity of AEs was graded according to National Cancer Institute-Common Toxicity Criteria for Adverse Events version 5.0.”* |
| **Organ Radiation Dosimetry** | “The organs that received the highest normalized absorbed doses...” – Conceptual error: Absorbed dose must be expressed in mGy/MBq or Gy/MBq; mSv/MBq is a unit of effective/equivalent dose. | We have revised the units for normalized absorbed dose to mGy/MBq. |
| **Tumor Dosimetry** | “all 10 patients (9 renal tumors and 1 external lymph node distant metastasis)” – Clarity/continuity error: Because right after it says “absorbed dose of a renal tumor and extrarenal lung distant metastasis could not be estimated”, that is, not all patients/tumors were actually analyzed. | While all patients were analyzed, 1 patient had 3 tumors (renal, lymph node, lung). Only the external lymph node distant metastasis in the patient who had 3 tumors could be analyzed. We have revised the wording for clarity:  *“Tumor dosimetry was performed in all 10 patients (9 renal tumors and 1 external lymph node distant metastasis). Two tumors were not evaluable in the 1 patient with 3 tumors”* |
|  | “due to limitations of the spherical model because the size of the renal tumor was >1000 g and the extrarenal tumor was <1 g” – Reproducibility error: does not provide technical criteria/reference, nor sufficient methodological basis to justify exclusion of data. | We have added references to the Methods for reproducibility. We have also added additional details in the Methods for clarity:  *“For specific tumor masses, the dose was calculated by interpolation using a power function to the previously determined volume of the lesion on the pre-screening CT or MRI images, assuming a density of 1.06 g/cm^3^.”* |
|  | “tumor normalized absorbed dose was significantly lower (0.28 [1.15] mGy/MBq [range: 0.12 to 0.31 mGy/MBq]; p=0.023).” – Context error: The result “p=0.023” appears isolated, without explicit mention of the test used in this comparison | The Mann-Whitney U test was used as stated in the Methods. We have revised the statement for accuracy:  *“In the 3 patients with non-ccRCC renal lesions, median (interquartile range) tumor normalized absorbed dose was significantly lower (0.31 [0.12-0.42] mGy/MBq; p=0.023).”* |
|  | “imaging results and 1 (TFE3 translocation RCC) had equivocal PET imaging result.” – Clarity/continuity error: The criterion for equivocality in the PET image is not explicitly defined (visual, quantitative/SUV, comparator), nor referenced. | [^89^Zr]Zr-girentuximab PET scan interpretation is based on visual examination only. We have added the following statement to the Methods:  *“Lesions of interest were considered PET positive or PET negative based on a visual scale, taking into account the background signal in surrounding organs and blood pool.”* |
| **PK** | The standard is to use “Pharmacokinetics” or “Pharmacokinetic analysis” as a subtitle, and never just “PK”. | We have revised the subtitle from “PK” to “Pharmacokinetics”. |
|  | Trough concertation was reached on Day 5. – Typo: The correct is “Trough concentration” | We have corrected the typo. |
|  | Insufficient description of methods for PK parameters | As stated in the Methods, whole blood PK parameters were calculated by non-compartmental analysis based on the actual sampling time relative to the start of administration using Phoenix WinNonlin 8.3. We have added deatils on PK parameters and calculations to the Supplemental Information. |
|  | Lack of detail for calculation of half-life and other PK parameters | We have added details on PK parameters and calculations to the Supplemental Information. |
| **Discussion** | “...support ability of 89 Zr-girentuximab PET to differentiate ccRCC from other renal lesions…”- Clarity/continuity error: it is not clear if there is sufficient quantitative data to support this statement (small sample, few non-ccRCC cases, poorly defined positivity definition). | We appreciate the reviewer’s concern regarding the strength of evidence for differentiating ccRCC from non-ccRCC lesions. We agree that our sample size, particularly the number of non-ccRCC cases, is limited. To address this, we have revised the text to the following:  *“Our findings provide preliminary evidence that [^89^Zr]Zr-girentuximab PET may help differentiate ccRCC from other renal lesions in Chinese patients, although confirmation in larger cohorts is warranted.”*  This revision acknowledges the potential of the tracer while clarifying that the current dataset is preliminary. |
|  | Lack of methodological detail on dosimetry analysis and comparison with previous studies | We have revised the statement in the Discussion section to include methodological details:  *“The renal tumor normalized absorbed dose range (0.12 to 5.41 mGy/MBq) overlapped with the ranges (1.90 to 11.6 mGy/MBq, and 0.67 to 6.55 mGy/MBq) reported in previously published studies calculated on OLINDA/EXM 2.1 and OLINDA, respectively (30,31).”* |
|  | “no AEs were observed..” – Typo: The correct would be “no AEs were observed.” | We have corrected the typo. |
| **Conclusion** | “theranostic approaches utilizing 89 Zr-girentuximab as a companion diagnostic are currently being explored.”- Reference error: There is no mention, method, or result about theranostics in the presented study. | We have removed this statement as suggested.  We agree that our study did not include theranostic interventions; however, the discussion section is intended to place our findings in a broader context and highlight potential future directions. [^89^Zr]Zr-girentuximab is currently under investigation for theranostic applications. To clarify this point, we have revised the statement as follows:  *“CAIX-targeted radiotracers, including [^89^Zr]Zr-girentuximab, are being actively investigated and may play an important role in future theranostic strategies for ccRCC.”* |
| **Table 1** | Blank lines between table sections (not allowed in international journals). | We have removed the blank lines in all tables. |
|  | Formatting of borders too thick/prominent for international standard. | We have reformatted tables to match journal guidelines. |
|  | Explanatory note (“Age is calculated...”) is outside the table footer format. | We have reformatted footnote to table footer format. |
|  | Age unit is not explicit in the data cell (“years” should accompany “57.2 (15.7)”). | We have added “years” to data cell for clarity. |
| **Table 2** | Inadequate formatting for international standard | We have reformatted tables to match journal guidelines. |
|  | Use of blank lines to separate groups within the table. Not allowed; should use grouping by subitems and/or italics/bold, never blank lines. | We have removed the blank lines in all tables. |
|  | Absence of severity grading for adverse events. There is no information about the grade/severity (Grade 1, 2, 3) of adverse events in the table, which is mandatory in clinical studies. | We have added severity grading for adverse events to Table 2. |
|  | Lack of clear specification of the criterion for classifying adverse events. It is not explicit in the table (or footnote) which system was used to classify adverse events (e.g., MedDRA, CTCAE). | We have added specifications to table and footnote on criterion for classifying adverse events. AEs were coded using MedDRA 26.0. The severity of AEs was graded according to National Cancer Institute-Common Toxicity Criteria for Adverse Events version 5.0. |
| **Table 3** | Incorrect unit (“Normalized Absorbed Dose” in mSv/MBq) “Absorbed dose” should be presented in mGy/MBq (or Gy/MBq), not in mSv/MBq. | We agree with your definition and have revised the units used throughout the manuscript for accuracy. |
|  | Inadequate visual separation (excess of lines and spacing). Many horizontal lines and artificial separation between parts of the table, not accepted by international journals. | We have removed the blank lines in all tables. |
|  | Lack of clarity in the footnote about “N = 10”*. The note “*N = 10 except where noted” is not sufficiently clear; it should be explicit for each organ (for example, “Uterus (n = 4)”) in a more detailed way. | We have removed the footnote. Table has been updated to indicate organ-specific sample sizes. |
| **Figure 1** | Inadequate intensity bar legend. Side bar is “SUV”, but there is no explanation about the normalization method (body weight? lean body mass?). | We have added the following explanation on the normalization method to the figure legend:  *“Standardized uptake values were normalized to body weight.”* |
| **Figure 2** | Y-axis without appropriate unit. It says “Mean ± SD (ng/mL)”. It is not explicit whether it is whole blood, plasma, radioactivity or immunologic concentration. | We have revised the y-axis in Figure 2 to include appropriate units. |

Finally, I must express my reservations as this is now a second recommendation for major revision. There is a real risk that the authors are either unable or unwilling to implement the necessary changes to bring the manuscript up to the required standard. While I am still inclined to offer the authors a further opportunity to revise, I believe rejection or transfer to a journal with less stringent requirements may also be reasonable alternatives, at your discretion.

In summary, while the scientific value of the study is not in question, the manuscript as currently presented still requires major revision. I would encourage the authors to carefully address each of the above points, making explicit changes in both the text and supporting materials, to meet the journal's standards for publication.

**Response:** We thank the reviewer for their constructive feedback and recognition of the study’s scientific value. We would like to clarify that this is our first round of peer review comments. We are fully committed to addressing all points raised to ensure the manuscript meets the journal’s standards.

**Reviewer #3 comments:**

1. (Abstract) - background should explain the innovation and meaning of this study. What's the problems the current Phase 3 study exist?

**Response:** We have added a short statement to the Abstract to address the unmet need:

*“However, [^89^Zr]Zr-girentuximab has not been studied in Chinese patients.”*

2. (Introduction) - background should provide more information of the current status of diagnosing and treatments in ccRCC. For example, is there any other PET agents used now and what's the pitfalls?

**Response:** We have added additional statements to the Introduction to address the current use of other PET agents for detection of primary ccRCC:

*“Positron emission tomography (PET) with commonly used tracers such as [^18^F]F-FDG is not a standard imaging modality in patients with ccRCC due to poor uptake, low specificity and sensitivity, and renal excretion (16, 24). PSMA PET tracers used in prostate cancer such as [^68^Ga]Ga-PSMA and [^18^F]F-PSMA have limited use in primary ccRCC due to variable PSMA expression in ccRCC, poor tumor visualization given high physiological PSMA uptake in surrounding renal tissue and renal elimination (25-27).”*

3. (Material and Method) the attenuation correction is more recommended instead of absorption correction.

**Response:** We have revised the terminology from “absorption” correction to “attenuation” correction.

4. (Results) - Please provide more details of Table 1. For example, how many newly suspected RCC patients and how many recurrent patients? What's the injection dosage of 89Zr-girentuximab? The values of important baseline clinical items indicating renal or liver functions?

**Response:** We have revised Table 1 to include additional relevant baseline characteristics. As stated in the safety section, the mean (SD) decay corrected activity administered was 36.43 (0.68) MBq, with a range from 35.52 to 37.76 MBq.

5. (Results) - Did the patients underwent matched 18F-FDG PET scanning? If so, please add the figures into Results part.

**Response:** No, the patients did not undergo matched [^18^F]F-FDG PET.

6. (Results) - one patient was reported with LN metastasis in this study, how is the typical MIP figures of LNM?

**Response:** Additional studies will evaluate [^89^Zr]Zr-girentuximab PET in patients with metastatic ccRCC. The uptake is usually lower intensity than the primary ccRCC lesion, but still clearly distinguishable from background in positive cases.

7. (Results) - how is the curve of 89Zr-girentuximab radioactivity in tumor, liver and other organs after injection? Please add those into Figure 2.

**Response:** We have added to Figure 2 the mean (SD) organ percentage injected activity-time plot.

8. (Discussion) - the PSMA PET has also been used in patients with renal tumors. What's the problems of PSMA imaging and what's the advantage of 89Zr-girentuximab?

**Response:** We have added the following statements regarding PSMA PET to the Introduction, focusing on PSMA PET for detection of primary ccRCC as per the intent of this study:

*“PSMA PET tracers used in prostate cancer such as [^68^Ga]Ga-PSMA and [^18^F]F-PSMA have limited use in primary ccRCC is due to variable PSMA expression in ccRCC, poor tumor visualization given high physiological PSMA uptake in surrounding renal tissue, and renal elimination (25-27)”*

9. (Discussion) - dose ethnicity of patients mater a lot in the management of renal cancer? Since for 89Zr-girentuximab, there are some phase-3 researches already, what's the innovation of this phase 1 study? More reasons are suggested to be added in Discussion.

**Response:** Our Phase 1 study provides novel data on the pharmacokinetics and dosimetry of [^89^Zr]Zr-girentuximab in Chinese patients, with results supporting initiation of the Phase 3 registrational study (currently enrolling) in Chinese patients. We have added the following statements to the Discussion:

*“In this study, 5 (71%) of the 7 patients with histologically confirmed ccRCC had tumor lesions visible on a total-body scanner at 4 hours after [^89^Zr]Zr-girentuximab administration; additional studies are needed to confirm the value of early-phase imaging with [^89^Zr]Zr-girentuximab PET.”*

*“…this study extends prior findings from the Phase 3 ZIRCON study by providing preliminary evidence in a Chinese patient population; the diagnostic utility of [^89^Zr]Zr-girentuximab will be further assessed in the Phase 3 ZIRCON-CP study. Overall, results contribute to the understanding of dosimetry, safety, and pharmacokinetics in Chinese patients, thereby addressing current evidence gaps and supporting broader generalizability of [^89^Zr]Zr-girentuximab PET across diverse patient group.”*
